# Effects of commonly used analgesics on sleep architecture: a topical review

**DOI:** 10.1097/j.pain.0000000000003201

**Published:** 2024-03-05

**Authors:** Hanna Antila, Tuomas O. Lilius, Vinko Palada, Terhi Lohela, Rae F. Bell, Tarja Porkka-Heiskanen, Eija Kalso

**Affiliations:** a Neuroscience Center, Helsinki Institute of Life Science, University of Helsinki, Finland; b SleepWell Research Program Unit, Faculty of Medicine, University of Helsinki, Finland; c Individualized Drug Therapy Research Program Unit, Faculty of Medicine, University of Helsinki, Finland; d Department of Pharmacology, Faculty of Medicine, University of Helsinki, Finland; e Department of Emergency Medicine and Services, Helsinki University Hospital and University of Helsinki, Finland; f Department of Physiology, Faculty of Medicine, University of Helsinki, Finland; g Department of Anaesthesiology, Intensive Care and Pain Medicine, Helsinki University Hospital and University of Helsinki, Finland; h Regional Centre of Excellence in Palliative Care, Haukeland University Hospital, Bergen, Norway

## 1. Introduction

Sleep is essential for health. It has an important bidirectional relationship with pain: Pain disrupts sleep, while poor sleep augments pain intensity and enhances spread to multiple sites.^2,79^ Anxiety, fear, and worry are often associated with sleep problems and chronic pain. Sleep problems are reported by two-thirds of pain clinic patients.^72^

The different stages of sleep serve various functions that aim to preserve the homeostasis of both body and mind. Insufficient sleep has negative effects on immune, metabolic, cardiovascular, and cognitive functions, as well as on emotional regulation. Increased understanding of the importance of sleep has inspired pain researchers to focus on sleep as a target for therapeutic interventions in patients with pain.^46^ In this context, it is important to be aware of the effects that commonly used analgesics have on sleep.

In this review, we provide a short summary of the physiology of sleep and how it is affected by pain. We focus on the effects of analgesics on sleep in patients with persistent pain and in healthy volunteers to address the purely pharmacological effects of analgesics on sleep. We also provide insight from preclinical animal studies. We have restricted the review to common analgesic drugs such as paracetamol, nonsteroidal anti-inflammatory drugs (NSAIDs), opioids, tricyclic and dual-action antidepressants, and gabapentinoids. Finally, we provide some thoughts about where future research in this field might go.

## 2. Sleep

### 2.1. Sleep structure and physiology

**Figure 1.**
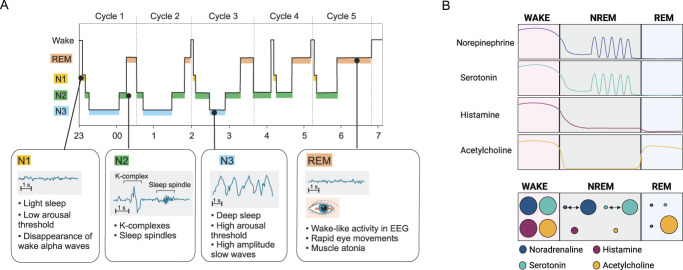
(A) Sleep stages and their characteristics. One sleep cycle includes different stages of non-rapid eye movement (NREM) sleep followed by REM sleep, and in humans, its average length is about 90 minutes.^19^ The transition from wakefulness to sleep happens through the N1 sleep stage, during which the occipital alpha (8-11 Hz) rhythm indicating the wake stage in humans starts to disappear in the EEG and slower theta waves (4-7.5 Hz) appear.^1,34^ The appearance of specific sleep microarchitectural features, K-complexes and sleep spindles, indicates transition to the N2 sleep stage. N2 is the predominant sleep stage and comprises about 50% of the total sleep time. Transitions to the N3 sleep and to REM sleep happen through N2 sleep. The deepest sleep stage with the highest arousal threshold is N3 sleep, characterized by slow waves, during which large populations of cortical neurons activate and deactivate in synchrony.^1^ During the night, the amount of deep N3 sleep is highest in the first sleep cycles and in the following cycles, its amount dissipates, whereas the amount of REM sleep increases toward the morning. In the EEG, REM sleep differs remarkably from NREM sleep resembling more the wake state; therefore, it is also called paradoxical sleep.^57^ Rapid eye movements and vivid dreaming occur during REM sleep, and muscle tone in skeletal muscles completely disappears. (B) NREM and REM sleep have different neurochemical environments in the brain. During REM sleep, noradrenergic, serotonergic, and histaminergic signaling are absent, while acetylcholine levels are simultaneously elevated. In NREM sleep, the levels of acetylcholine, histamine, norepinephrine, and serotonin are low, but noradrenergic and serotonergic neurons can show rhythmic, fluctuating activity.^6,27,58,61,69,81^ Despite the different levels during NREM and REM sleep, norepinephrine, serotonin, histamine, and acetylcholine are present at the highest levels during wakefulness and are able to promote cortical activation.^69^ (B) Created with BioRender.com.

Sleep is a dynamic process consisting of alternations between rapid eye movement (REM) sleep and non-REM (NREM) sleep. NREM sleep is further divided into 3 stages, N1, N2, and N3, which can be differentiated by their increasing threshold to arousal and characteristic electroencephalogram (EEG) oscillatory features. The sleep structure, stages, and their characteristics are presented in more detail in Figure 1A.

The timing of sleep is controlled by a circadian and the duration by a homeostatic process.^12^ The master oscillator in the suprachiasmatic nucleus (SCN) creates an endogenous circadian rhythm of about 24 hours.^82^ Most physiological functions, including sleep, are connected to this rhythmicity, either by directly adhering to the endogenous oscillator or indirectly as diurnal rhythms. Failure to conform to this rhythmicity will create physiological dysfunction and is one of the most important factors leading to sleep problems.

Homeostatic control of sleep entails an extended period of wakefulness followed by a prolonged, intensified sleep period, often manifested by increase in N3 sleep.^12^ The key concept of sleep homeostasis is the sleep pressure that accumulates during waking and initiates sleep. Several mediators of sleep pressure have been proposed, for example, adenosine, increased number or size of synapses in the brain, and accumulation of metabolites associated with wakefulness.^86^ The glymphatic system, a brain-wide perivascular pathway that is most active during deep sleep, seems to play an important role in facilitating the clearance of metabolic products accumulated in the brain during wakefulness.^53,109^

Sleep and the immune system are bidirectionally connected. Some proinflammatory cytokines such as TNF-α and interleukin-1, as well as prostaglandin D2 can have sleep promoting effects.^80,101^ The increased sleep observed in response to infection can be beneficial by augmenting immune system function, whereas sleep loss per se can induce an inflammatory state and impair immune response.^10,80^

In the brain, sleep has an essential role in plasticity, memory consolidation, and cognition. Sleep spindles, the characteristic oscillations during N2 sleep, as well as N3 sleep slow waves and REM sleep have been associated with memory consolidation and learning.^15,32,100^ Sleep, in particular REM sleep, also seems to be important for emotional regulation.^42^ For instance, uninterrupted REM sleep episodes can afford protection from amygdala overactivation associated with anxiety.^106^

During sleep, notable physiological changes occur. In NREM sleep, body temperature, heart rate, blood pressure, and breathing rate decrease, whereas in REM sleep, their control is more irregular.^86^ Changes in the brain neurochemical environment between wake, NREM, and REM sleep can explain some of the physiological and functional differences observed in these distinct brain states (Fig. 1B).

### 2.2. Sleep disturbances and pain

The effects of sleep disturbances on pain sensitivity are well conserved across species, but sex differences exist as female patients develop more hypersensitivity than male patients.^63^ Disturbed sleep can mediate pain hypersensitivity at both sensory and emotional levels of the pain system.

Sleep loss promotes a proinflammatory effect that can aggravate pain complaints.^43,76^ Elevated levels of proinflammatory cytokines can amplify nociceptive signaling, for example, by increasing the sensitivity of nociceptors or by affecting the mesolimbic system.^51,54,107^ The mesolimbic system has an important role in determining the salience of the painful stimulus and modulating the reward system.^112^ Consequently, sleep loss-induced dysregulation of this system can change the salience of the pain experience or its expectation.^95,103^ The core brain regions of the mesolimbic system, ventral tegmental area and nucleus accumbens, are also involved in the regulation of sleep-wake behaviours.^22,29,77,113,114^

It is challenging to discriminate the function of different sleep stages on pain sensitivity, but evidence highlights the importance of N3 slow wave sleep. Restoring the amount of N3 sleep after sleep deprivation elevates pain threshold, whereas experimental disruption of slow wave sleep decreases pain threshold and increases musculoskeletal discomfort in healthy volunteers.^68,73,78^ In addition, decrease in N3 sleep has been observed in patients with neuropathic pain.^11,31^ Neuropathic pain involves a neuroinflammatory element defined by pathological glial activation and release of neuroinflammatory cytokines to the extracellular space.^56^ Because the glymphatic system is most active during deep sleep, one important question is whether pain-induced sleep reduction disturbs glymphatic flow and affects pain chronification by inhibiting clearance of cytokines. Indeed, a preclinical study has shown that acute nociceptive stimulation hinders tracer clearance from deep brain structures.^97^

Shorter duration of N2 sleep has been linked to higher pain intensity in patients with fibromyalgia.^16^ The occurrence and characteristics of sleep spindles, typical of N2 sleep, show individual variability. A reduced amount of spindles has been observed in patients with fibromyalgia and in rats with inflammatory pain.^17,66^

The role of disturbed REM sleep and pain is complex because it would most likely involve the mesolimbic processes associated with emotion and cognition. It has been suggested that loss of REM sleep could induce hyperalgesia the following day. However, on the whole, it seems that the main driver for pain hypersensitivity is the significant total sleep loss over time, rather than specific loss of REM sleep.^63,87^

## 3. The effects of analgesics on sleep

A simple search of PubMed was conducted using the terms “polysomnography”, “EEG”, and “sleep” combined with the analgesic or analgesic group names. After screening, studies where sleep parameters were reported using polysomnography (PSG) were included for consideration. Studies where sleep was only assessed using questionnaires or other subjective measures were excluded. The selected studies evaluated drug effects on sleep in patients with pain but also in healthy adult volunteers, to avoid the confounding effects of pain. The patient groups included those with fibromyalgia, dysmenorrhea, low back pain, osteoarthritis, and painful diabetic polyneuropathy. The small number of subjects in most studies and the heterogeneity of study designs precluded meta-analysis. Detailed information about the studies can be found in Table 1.

**Table 1:** Effects of analgesics on sleep structure—detailed information of the studies.

Treatment		Subjects and study design			Sleep parameters								PAIN
Drug and dose reference	Treatment duration	Subjects	Design		N1 drug X̅ ± SD placebo X̅ ± SD	N2 drug X̅ ± SD placebo X̅ ± SD	N3 drug X̅ ± SD placebo X̅ ± SD	REM drug X̅ ± SD placebo X̅ ± SD	WAKE drug X̅ ± SD placebo X̅ ± SD	TST drug X̅ ± SD placebo X̅ ± SD	Sleep efficiency drug X̅ ± SD placebo X̅ ± SD	WASO drug X̅ ± SD placebo X̅ ± SD	
**NSAIDs and paracetamol, healthy volunteers**													
Paracetamol 650 mg^74^	1 d, drug given at night	9 drug, 10 placebo, F/M	RCT, placebo-controlled		ns	ns	ns	ns	ns		ns	ns	
ASA 650 mg^74^	1 d, drug given at night	9 drug, 10 placebo, F/M	RCT, placebo-controlled		ns	ns	ns	ns	**↑** *P* < 0.05 9.0 ± 2.4% 8.0 ± 0.6%		**↓** *P* < 0.05 91.0 ± 2.4% 96.2 ± 0.6%		
ASA 600 mg x3^50^	4 d	8 drug, 8 placebo, F	RCT, placebo-controlled			**↑** *P* ≤ 0.01 197 ± 22.6 min 170 ± 20.5 min	**↓** *P* ≤ 0.01 88 ± 22.5 min 109 ± 20.0 min	ns	ns	ns			
Ibuprofen 400 mg^74^	1 d, drug given at night	9 drug, 10 placebo, F/M	RCT, placebo-controlled		ns	ns	ns	ns	**↑** *P* < 0.05 12.5 ± 2.1% 3.8 ± 0.6%		**↓** *P* < 0.05 87.5 ± 2.1% 96.2 ± 0.6%		
Ibuprofen 400 mg x3^40^	1 d, (drug given 3 pm, 7 pm, 11 pm)	15 drug, 15 placebo	RCT, placebo-controlled		ns	ns	ns	ns	ns		ns	ns	
**NSAIDs and paracetamol, patients**													
Paracetamol 1300 mg x3^8^	1 d (5 hours before sleep, lights off, morning)	16 healthy or dysmenorrhea, F		RCT, placebo-controlled, crossover	ns	ns	ns	ns	ns	ns			Not assessed
Diclofenac 50 mg x3^52^	1 d (last 30 minutes before bedtime)	15 patients with dysmenorrhea pain, F		RCT, placebo-controlled, crossover	**↓** *P* < 0.05 4 ± 1% 6 ± 3%	ns	ns	**↑** *P* < 0.01 26 ± 3% 22 ± 5%	ns	ns	**↑** *P* < 0.05 97 ± 1% 95 ± 3%	ns	100 mm VAS ↓ *P* < 0.05

Drug	Duration	Subjects	Design	N1	N2	N3	REM	WAKE	TST	SE	WASO	PAIN
**Opioids and tramadol, healthy volunteers**												
Morphine sulphate 15 mg (sustained release), p.o^25^	1 d	42	RCT, placebo-controlled, crossover	ns	**↑** *P* < 0.05 61.3 ± 1.1% 58.5 ± 1.4%	**↓** (N3 + N4) *P* < 0.05 7.5% 11.6%	ns		ns	ns	ns	
Morphine sulphate 0.1 mg/kg, i.v^96^	1d (30-60 minutes bef. lights off and 3-4 am)	7, F/M	RCT, placebo-controlled, crossover	ns	**↑** *P* = 0.001 70.3 ± 3.9% 55.1 ± 3.8% (SEM)	**↓** *P* = 0.0027 5.5 ± 2.9% 16.8 ± 2.0% (SEM)	**↓** *P* = 0.046 15.6 ± 2.5% 21.8 ± 2.4% (SEM)		ns	ns	ns	
Tramadol 50 mg^104^	1 d (evening)	8, F/M	RCT, placebo-controlled, crossover	ns	**↑** *P* = 0.03	Stage 4 ↓ *P* = 0.04	ns					
Tramadol 100 mg^104^	1 d (evening)	8, F/M	RCT, placebo-controlled, crossover	ns	**↑** *P* = 0.02	Stage 4 ↓ *P* = 0.02	**↓** *P* = 0.005					
Methadone 5 mg, p.o^25^	1 d	42	RCT, placebo-controlled, crossover	ns	**↑** *P* < 0.05 63.8 ± 1.3% 58.5 ± 1.4%	**↓** (N3 + N4) *P* < 0.05 7.1% 11.6%	ns		ns	ns	ns	
**Opioids and tramadol, patients**												
Different opioids^23^		Patients with fibromyalgia with insomnia, 65 opioid users, 128 nonusers, F: 94%	Opioid vs nonopioid users	ns	**↑** *P* =0.04 B = 1.39 ± 0.71% (SE)	**↓** *P* = 0.002 B = −4.90 ± 1.53% (SE)	ns		ns	ns	ns	Scale 0-100
Morphine sulphate 30 mg (extended release)^89^	14 d (morning)	10 sleep disturbances + osteoarthritis F/M	Single-blind, placebo-lead-in		**↑** *P* ≤ 0.05 296.7 min 260.5 min	ns	ns		**↑** *P* ≤ 0.05 422.9 min 385.6 min	ns	ns	BPI ↓ *P* < 0.05
Morphine sulphate 60 mg (extended release)^89^	14 d (morning)	12 osteoarthritis + sleep disturbances, F/M	Single-blind, placebo-lead-in		ns	ns	ns		ns	ns	ns	BPI ↓ *P* < 0.05

Drug	Duration	Subjects	Design	N1	N2	N3	REM	WAKE	TST	SE	WASO	PAIN
**Tricyclic and SNRI antidepressant drugs, healthy volunteers**												
Amitriptyline 50 mg^108^	4 d (PSG at night 3)	19 drug, 20 placebo, F/M	RCT, placebo-controlled	ns	**↑** *P* < 0.001 54.2 ± 5.8% 45.7 ± 5.4%	ns	**↓** *P* < 0.001 12.9 ± 3.4% 22.6 ± 4.8%	ns	ns	ns	ns	
Amitriptyline 75 mg^26^	1 d	14, M	RCT, placebo-controlled, crossover	ns	**↑** *P* < 0.001 64.3 ± 6.9% 53.2 ± 4.1%	ns	**↓** *P* < 0.001 8.6 ± 4.7% 21.4 ± 3.4%	ns	ns	Ns	**↓** *P* = 0.045 26.3 ± 18.8 min 31.2 ± 13.6 min	
Amitriptyline 50 mg^45^	28 d (20 minutes before bedtime)	14, M	Placebo-controlled, crossover	ns	**↑** *P* < 0.01 41.6 min compared with placebo*	ns	**↓** *P* < 0.001 60 min compared with placebo*		ns			
Amitriptyline 75 mg^41^	1 d (25 mg 9:30 pm + 50 mg 1:30 am)	13 drug, 15 placebo, M	RCT, placebo-controlled	ns	**↑** *P* = 0.001 61.4 ± 10.1% 46.5 ± 8.1%	ns	**↓** *P* < 0.001 4.1 ± 3.5% 16.9 ± 6.1%		**↑** *P* = 0.02 438.5 ± 32.8 min 410.1 ± 38.2 min	**↑** *P* = 0.04 91.9 ± 6.9% 86.1 ± 8.1%	**↓** *P* = 0.02 4.3% ± 1.6 9.5% ± 7.0	
Imipramine 40 mg^111^	1 d total 40 mg: 9 pm (−1), 8 am, 12 pm, 6 pm, 9 pm	8, M	RCT, single-blind, placebo-controlled, crossover		**↑** +N1 *P* < 0.002 78.7 ± 5.2% 63.6 ± 7.7%	ns	**↓** *P* = 0.0007 12.3 ± 3.3% 22.3 ± 7.3%		ns	ns		
Desipramine 50 mg x2^20^	7 d	12, M	RCT, placebo-controlled, crossover	ns	ns	ns	**↓** *P* < 0.0001 43.5 ± 22.6 min 103.6 ± 17.7 min		**↓** *P* = 0.005 349.9 ± 82.0 min 411.7 ± 37.1 min	ns	ns	
Duloxetine 80 mg (morning)^20^	7 d (PSG day 6)	6, M	RCT, placebo-controlled, crossover	ns	**↑** *P* = 0.004 271 ± 34.6 min 209.3 ± 32.8 min	ns	**↓** *P* < 0.0001 44.3 ± 21.7 min 103.6 ± 17.7 min		ns	ns	ns	
Duloxetine 60 mg x2^20^	7 d (PSG day 6)	6, M	RCT, placebo-controlled, crossover	ns	**↑** *P* = 0.001 281.4 ± 47.3 min 209.3 ± 32.8 min	Stage 4 **↓** *P* = 0.013 27.1 ± 24.7 min 54.2 ± 21.2 min	**↓** *P* < 0.0001 26.6 ± 10.4 min 103.6 ± 17.7 min		ns	ns	ns	
Venlafaxine 75 mg 2 d + 150 mg 2 d^92^	4 d (evening, 1 hour after sleep recording started)	8, F/M	Compared with baseline	**↑** *P* < 0.0001 148.6 ± 40.4 min 26.3 ± 9.2 min	**↓** *P* < 0.007 121.1 ± 57.3 min 212.0 ± 26.9 min	**↓** *P* < 0.009 10.9 ± 10.6 min 35.2 ± 9.9 min (stage 4 ns)	**↓** *P* < 0.00001 0.0 ± 0.0 min 31.0 ± 9.1 min	**↑** *P* < 0.0007 161.8 ± 65.3 min 29.4 ± 18.4 min				
**Tricyclic and SNRI antidepressant drugs, patients**												
Amitriptyline 25 mg x2 for 14d and 25 mg + 50 mg for 14d^14^	28 d (opioids, NSAIDs, paracetamol allowed)	23 painful diabetic polyneuropathy, F/M	RCT, placebo-run-in	NREM **↑** *P* < 0.0001 343.6 ± 9.3 min 291.6 ± 7.4 (SE)			**↓** *P* < 0.0001 50.2 ± 5.4 min 77.0 ± 5.2 min (SE)		ns	ns	**↓** *P* < 0.05 66.6 ± 10.8 min 91.0 ± 9.4 min (SE)	BPI ns
Amitriptyline 25 mg^18^	8 wk (bef. bedtime) on/off + 8 wks off/on	22, fibromyalgia, F: 95.5%	RCT, placebo-controlled Crossover	**↓** *P* ≤ 0.05 8.14 ± 4.2% 5.55 ± 2.8%	ns	ns	ns		ns			10 cm VAS ↓ *P* < 0.05
Duloxetine 60 mg x1 for 14 d and 60 mg x2 for 14 d^14^	28 d (opioids, NSAIDs, paracetamol allowed)	23, painful diabetic polyneuropathy F/M	RCT, placebo-run-in	NREM **↑** *P* < 0.05 326.7 ± 12.6 min 298.0 ± 9.6 min (SE)			**↓** *P* < 0.0001 29.9 ± 5.7 min 83.4 ± 7.5 min (SE)		**↓** *P* < 0.05 356.6 ± 13.8 min 381.4 ± 9.4 min (SE)	**↓** *P* < 0.05 74.2 ± 2.9% 79.4 ± 2.0% (SE)	ns	BPI ns

Drug	Duration	Subjects	Design	N1	N2	N3	REM	WAKE	TST	SE	WASO	PAIN
**Gabapentinoids, healthy volunteers**												
Gabapentin 600 mg x3^35^	Titration to 1800 mg for 6d, PSG 7-10 later	10 drug, 9 control, F/M	Compared with baseline	ns	ns	**↑** *P* = 0.007 13.0 ± 0.07% 8.0 ± 0.03%	ns		ns	ns	ns	
Gabapentin 300 mg^85^	1 d	8 community-dwelling older men	RCT, placebo-controlled, crossover	ns	ns	ns	ns		ns	ns	ns	
Pregabalin 150 mg x3^47^	3d	24, M	RCT, placebo-controlled Crossover	**↓** *P* < 0.001 Last day 3.1 ± 2.1% 5.3 ± 2.5%	**↓** *P* < 0.05 Last day 39.0 ± 7.2% 39.8 ± 8.0%	**↑** N4: *P* < 0.001 Last day N3 + N4 36.4% 25.3%	ns		**↑** *P* < 0.001 Last day: 458 ± 10.3 min 432.7 ± 22.4 min	**↑** *P* < 0.001 Last day: 95.3 ± 2.1% 90.1 ± 4.7%		
Gabapentin 250 mg^88^	1 d	127 placebo, 125 drug, sleep phase advance 5 hours, M/F	RCT, placebo-controlled	**↓** *P* ≤ 0.001 11.8 ± 0.7% 15.1 ± 1.0% (SE)	ns	**↑** *P* ≤ 0.05 15.4 ± 1.0% 12.6 ± 0.9% (SE)	ns		**↑** *P* ≤ 0.001 356.5 ± 7.3 min 311.4 ± 8.4 min (SE)		**↓** *P* ≤ 0.001 100.7 ± 5.8 min 135.7 ± 7.0 min (SE)	
Gabapentin 500 mg^88^	1 d	127 placebo, 125 drug, sleep phase advance 5 hours, M/F	RCT, placebo-controlled	**↓** *P* ≤ 0.001 10.8 ± 0.7% 15.1 ± 1.0% (SE)	ns	**↑** *P* ≤ 0.001 17.0 ± 1.1%, 12.6 ± 0.9% (SE)	ns		**↑** *P* ≤ 0.001 378.7 ± 7.3 min 311.4 ± 8.4 min (SE)		**↓** *P* ≤ 0.001 73.2 ± 5.8 min 135.7 ± 7.0 min (SE)	
Gabapentin 250 mg^37^	28 d	115 placebo, 122 drug, acute sleep phase advance 5 hours, F/M	RCT, placebo-controlled	ns	ns	ns	**↑** *P* ≤ 0.05 15.6 ± 0.5% 13.6 ± 0.6% (SE)		**↑** *P* ≤ 0.001 335.3 ± 8.2 min 289.1 ± 10.2 min (SE)		**↓** *P* ≤ 0.001 113.6 ± 8.1 min 152.3 ± 9.3 min (SE)	
**Gabapentinoids, patients**												
Pregabalin 150 mg x2 for 14d and 300 mg x2 for 14 d^14^	28 d (opioids, NSAIDs, paracetamol allowed)	19 painful diabetic polyneuropathy, F/M	RCT, placebo-lead-in	NREM ↑ *P* < 0.0001 348.3 ± 10.1 min 291.5 ± 10.6 min (SE)			**↓** *P* < 0.01 62.0 ± 6.9 min 80.1 ± 6.0 min (SE)		**↑** *P* < 0.01 410.3 ± 10.2 min 371.6 ± 11.8 min (SE)	**↑** *P* < 0.01 85.4 ± 2.1% 77.3 ± 2.5% (SE)	**↓** *P* < 0.01 57.2 ± 10.3 min 90.9 ± 11.8 min (SE)	BPI ns
Pregabalin 300-450 mg^90^	4 wks, including titration max 14 d	115 patients with fibromyalgia, F:87%	RCT, placebo-controlled, crossover			**↑** *P* = 0.0024 17.2 ± 1.0% 15.0 ± 1.0%			**↑** *P* < 0.0001 396.2 ± 4.7 min 370.6 ± 4.7 min	**↑** *P* < 0.0001 82.6 ± 1.0% 77.2 ± 1.0%	**↓** *P* < 0.0001 51.5 ± 3.8 min 70.7 ± 3.8 min	Scale 0-10 ↓ *P* = 0.0084

**↑**, increase; **↓**, decrease; ASA, acetyl salicylic acid; BPI, brief pain inventory; F, female; i.v., intravenous; M, male; NSAID, nonsteroidal anti-inflammatory drug; p.o., per os (oral administration); PSG, polysomnography; RCT, randomized controlled trial; SD, standard deviation; SE, standard error; SEM, standard error of mean; TST, total sleep time; VAS, visual analogue scale; WASO, wake after sleep onset; ns, not significant, X̅, mean.

### 3.1. The effects of analgesics on sleep stages

#### 3.1.1. Nonsteroidal anti-inflammatory drugs and paracetamol

Nonsteroidal anti-inflammatory drugs convey their analgesic effect by inhibiting the cyclo-oxygenase (COX) enzymes COX-1 or COX-2, depending on their selectivity. Cyclo-oxygenase-2 converts omega-6 arachidonic fatty acid to prostaglandins that sensitize both peripheral and central neurons.^84^ The mechanism by which paracetamol alleviates pain has not yet been determined, but central COX inhibition and modulation of the endocannabinoid system have been suggested to be involved.^7^

Ibuprofen and paracetamol did not induce notable alterations in sleep architecture after acute (1 day) treatment in healthy volunteers but ibuprofen impaired sleep efficiency by increasing the amount of wakefulness.^40,74^ Acetylsalicylic acid (ASA) increased N2 sleep and decreased N3 sleep in healthy volunteers during 4 days of administration; however, these effects were not observed in a study where only one dose of ASA was administered before bedtime.^50,74^ In patients with dysmenorrhea, diclofenac increased REM sleep compared with placebo, whereas paracetamol did not significantly affect sleep.^8,52^

#### 3.1.2. Opioids

The endogenous opioid peptides control or modify several physiological functions, including pain and vigilance. Opioids convey most of their effects by activating the opioid receptors MOP, DOP, KOP, and NOP (ORL1).^60^ The analgesic effects are mediated by the MOP receptors and the sedative effects by the MOP and KOP receptors.^55^ Long-term opioid administration can significantly weaken the endogenous opioid system and thereby also its effects on sleep regulation. Opioid use can lead to sleep-disordered breathing, central apnea, upper airway obstruction, and hypoxemia.^36^

Unlike other analgesics, opioids consistently decreased deep N3 sleep in healthy volunteers.^25,96,104^ The amount of N2 sleep, on the contrary, was increased. In some studies, also the amount of REM sleep was reduced.^96,104^ The amount of wake after sleep onset (WASO) was not affected by acute opioid treatment in healthy volunteers or by chronic opioid treatment in patients. The sleep effects of long-term opioid treatment in patients with fibromyalgia and patients with osteoarthritis resemble those observed in healthy volunteers. However, in patients with pre-existing sleep disturbances, the amount of N3 sleep was not further reduced.^23,89^

The response to opioids might differ depending on the dose and treatment duration. Especially in animal studies, opioids can cause agitation or wakefulness rather than sleep.^5,24,28,39,105^

#### 3.1.3. Tricyclic and dual-action antidepressant drugs

Tricyclic antidepressants are efficacious in the treatment of neuropathic pain at lower doses than those needed to treat depression.^33,70^ Amitriptyline and its metabolite nortriptyline have serotonergic, noradrenergic, anticholinergic, and antihistaminergic effects. The dual-action antidepressants duloxetine and venlafaxine inhibit the reuptake of both serotonin and norepinephrine (SNRI), the latter effect being essential for their efficacy in neuropathic pain. Antidepressant drugs increase descending inhibition to the spinal cord, which is considered their main analgesic mechanism.^71^ Serotonin and norepinephrine antidepressants also have anxiolytic properties which can indirectly reduce the pain experience.^13,91^

The most remarkable effect of tricyclic and SNRI antidepressant drugs on sleep is reduction of REM sleep, demonstrated by all the compounds that belong to the class of antidepressant drugs in the examined studies.^14,20,26,41,62,67,92,108,111^ The REM-suppressing effect of antidepressant drugs has also consistently been found in preclinical studies.^59,75,93,94^ Total sleep time and N1 or N3 sleep stages were not invariably affected. Rather, the REM sleep reduction seemed to be compensated by an increased amount of N2 sleep. Particularly, treatment with tricyclic antidepressants seemed to maintain the amount of N3 sleep. These compensatory increases in NREM sleep preserve the total sleep time, which is more important for pain sensitivity than a reduction in REM sleep.^63^ The tricyclic antidepressant used in most studies was amitriptyline. It has antihistaminergic properties and has sedative effects already at the low doses commonly used in chronic pain and is administered in the early evening hours.

In healthy volunteers, administration of the SNRI antidepressant venlafaxine in the evening for 4 days reduced the amount of N2 and N3 sleep.^92^ However, the comparison was made with baseline measurements and not with placebo. After 6 days of treatment, another SNRI antidepressant duloxetine did not alter N3 sleep when administered in a dose of 80 mg in the morning. However, when duloxetine 60 mg was administered twice daily, the amount of deep sleep was reduced after 6 days of treatment.^20^ These results could indicate that SNRI antidepressants negatively affect deep sleep, especially when administered close to bedtime.

#### 3.1.4. Gabapentinoids

Gabapentin and pregabalin bind to the α_2_δ_1_-subunit of the voltage-gated calcium channels and reduce the release of pronociceptive neurotransmitters including glutamate.^21^ In addition to being efficacious in neuropathic pain, both drugs have anxiolytic effects which may also enhance analgesia.^49^ Pregabalin has been shown to improve sleep in patients with generalized anxiety disorder.^48^

When administered to healthy volunteers and patients with fibromyalgia, gabapentinoids increased N3 sleep and total sleep time, while reducing light N1 sleep and WASO.^47,88,90^ When pregabalin was administered long-term to patients with painful diabetic polyneuropathy, NREM sleep was increased.^14^ Unfortunately, the NREM stage-specific effects were not described. In some studies, gabapentinoids seemed to reduce N2 sleep or increase REM sleep, but these effects were not as consistent as the N3-increasing effect.^9,14,47,88^ Only 1 study, conducted on community-dwelling older men receiving a single dose of gabapentin, did not detect any effects on sleep structure.^85^ Supporting the clinical findings, gabapentinoids have increased NREM sleep amount and duration in animal studies, as well as restored NREM sleep in a mouse model of neuropathic pain.^30,64,99^

### 3.2. Interactions between circadian rhythms and analgesics

Circadian rhythms influence the efficacy of analgesics by affecting both pharmacokinetics (absorption, distribution, metabolism, excretion) and pharmacodynamics (intracellular signaling, target molecules, gene transcription), suggesting that time-targeted analgesic administration could optimize drug efficacy and safety.^82^ This is particularly evident for NSAIDs, opioids, antidepressants, and gabapentinoids that have downstream targets or receptors showing circadian rhythmicity.^65,98,110^ Analgesics can also directly interfere with circadian rhythmicity by altering the expression of core circadian genes.^3,38,102^

## 4. Conclusions and future directions

Disturbed sleep is increasingly recognized as a key factor in the development and maintenance of chronic pain and is considered an important target for treatment.^46^ Sleep problems and pain share a number of comorbidities such as anxiety, stress, and depression, resulting in a vicious circle where independent factors may reinforce each other. To break this circle, the contributing factors in the individual patient need to be identified to optimize the treatment, including pharmacological interventions.

Polysomnography is the preferred research tool, but only few studies have used it to assess the effects of analgesics on sleep in patients with pain. There is generally a paucity of information concerning the effects of analgesics on sleep, especially regarding long-term use. This is the case for even the most commonly used analgesics such as NSAIDs.

Opioids seem to have the most deleterious effects on sleep because they reduce deep N3 sleep. Opioids can also cause sleep-disordered breathing and respiratory depression, providing further argumentation for limiting their use in chronic noncancer pain.

Tricyclic antidepressants or gabapentinoids seem to have beneficial effects on both sleep and pain. How much the improved sleep contributes to the analgesic effect of these drugs is not known. Gabapentinoids and SNRI antidepressants also have anxiolytic effects which may indirectly contribute to improved sleep and pain.

The effects of disturbed glymphatic clearance on pain chronification is an important topic for future research. Preclinical studies suggest that glymphatic clearance is under circadian control and is enhanced during slow wave sleep or anaesthesia, during which noradrenergic tone is low.^44,53,109^ The sedative α_2_-adrenergic agonist dexmedetomidine reduces central norepinephrine levels and induces cortical slow waves and has been shown to promote glymphatic flow.^83^ How other drugs that target the noradrenergic system, such as SNRI antidepressants, affect glymphatic clearance needs to be elucidated. An interesting question is also whether promoting sleep with other pharmacological agents, such as melatonin, could alleviate pain. A recent systematic review concluded that only low-quality evidence is available on this topic.^4^

## Conflict of interest statement

Eija Kalso has received lecture fees from GSK and Haleon and financial compensation for advisory board work from Pfizer and Orion Pharma, unrelated to this work. Other authors report no conflicts of interest.
